# Identifying Important Pairwise Logratios in Compositional Data with Sparse Principal Component Analysis

**DOI:** 10.1007/s11004-024-10159-0

**Published:** 2024-10-10

**Authors:** Viktorie Nesrstová, Ines Wilms, Karel Hron, Peter Filzmoser

**Affiliations:** 1 Department of Mathematical Analysis and Applications of Mathematics, Palacký University Olomouc, Faculty of Science, 17. listopadu 12, Olomouc, Czech Republic; 2 Department of Informatics and Quantitative Methods, Faculty of Informatics and Management, University of Hradec Králové, Hradecká 1249/6, Hradec Králové, Czech Republic; 3 Department of Quantitative Economics, Maastricht University, Tongersestraat 53, Maastricht, The Netherlands; 4 Institute of Statistics and Mathematical Methods in Economics, TU Wien, Wiedner Hauptstraße 8-10, Vienna, Austria

**Keywords:** Compositional data, Pairwise logratios, Sparse PCA, Geochemical data

## Abstract

Compositional data are characterized by the fact that their elemental information is contained in simple pairwise logratios of the parts that constitute the composition. While pairwise logratios are typically easy to interpret, the number of possible pairs to consider quickly becomes too large even for medium-sized compositions, which may hinder interpretability in further multivariate analysis. Sparse methods can therefore be useful for identifying a few important pairwise logratios (and parts contained in them) from the total candidate set. To this end, we propose a procedure based on the construction of all possible pairwise logratios and employ sparse principal component analysis to identify important pairwise logratios. The performance of the procedure is demonstrated with both simulated and real-world data. In our empirical analysis, we propose three visual tools showing (i) the balance between sparsity and explained variability, (ii) the stability of the pairwise logratios, and (iii) the importance of the original compositional parts to aid practitioners in their model interpretation.

## Introduction

Compositional data (CoDa) are data that carry relative information in their parts (components) (Aitchison 1986). One can typically think of multivariate observations measured in units such as percentages or proportions which result in a constant sum (100 or 1, respectively) of their parts. However, the sum of their parts is in fact irrelevant; importantly, one should think of CoDa as scale-invariant objects (Pawlowsky-Glahn et al. 2015). Seminal information in CoDa is contained in the pairwise logratios (PLRs) between the parts of the composition. Even though simple PLRs are often preferred in multivariate analysis thanks to their straightforward interpretation (as demonstrated in applications; see, e.g., Greenacre, 2018, 2019), they quickly create a curse of dimensionality. Indeed, considering a composition of *D* parts, the number of possible PLRs to consider is $D(D-1)$. This number rapidly increases even for medium-sized compositions, which in turn complicates interpretability in further multivariate analysis. It is therefore desirable to identify a few PLRs that are most important (relevant) for the data structure. In this paper, we propose a sparse principal component analysis method for identifying such PLRs.

The relative nature of CoDa prevents standard statistical methods from being applied directly. This can, however, be easily overcome by expressing compositions in real logratio coordinates. A popular choice in this context is orthonormal logratio coordinates (olr), with the special case of balance coordinates being promoted in recent years (Pawlowsky-Glahn et al. 2015). These coordinates are constructed such that they express the dominance of one group of compositional parts with respect to another group. They are built based on the procedure called sequential binary partition (SBP), where non-overlapping groupings of parts are gradually derived. However, to build a meaningful SBP, prior expert knowledge of the data is usually required. But such knowledge could be particularly complicated to acquire for high-dimensional compositions, as the dimensionality affects both the construction of the SBP and interpretation of balance coordinates. While there exist data-driven algorithms to overcome this obvious handicap (Martín-Fernández et al. 2018), an appealing alternative is to analyze CoDa through simple PLRs that typically greatly facilitate interpretability.

Since the dimensionality of a *D*-part composition is only $D-1$ (Pawlowsky-Glahn et al. 2015), analyzing all PLRs simultaneously results in a positive semidefinite covariance matrix, which causes problems and possible inconsistencies in many statistical methods. Fortunately, this is not the case (among others) for principal component analysis (PCA), which is also one of most commonly used methods for multivariate data analysis in the compositional context (see, e.g., Filzmoser et al., 2018; Greenacre, 2018 for textbook introductions on the use of PCA for CoDa across various application fields ranging from geosciences and “omics” fields, including genomics, proteomics, and metabolomics, to chemometrics), mostly due to its simplicity and straightforward interpretation. Alternative dimension reduction methods would include partial least squares, principal balances, and robust pairwise logratios (rPLR) based on two groups of observations (e.g, chapter 11 in Filzmoser et al., 2018, and Walach et al., 2017), or more recent developments such as the PARAFAC model of Di Palma et al. (2018) for three-way CoDa. For (standard) PCA, using either all PLRs or any olr coordinates (or centered logratio coefficients, probably the most popular in this context, see Aitchison and Greenacre, 2002) leads to the same score values, and therefore also the same loadings representing PLRs (Daunis-i Estadella et al. 2011; Hron et al. 2021). Using all PLRs directly as a proper representation of compositions is also preferred when affine equivariance (or even just orthogonal equivariance, as for PCA) is lacking; see, for example, Alfons et al. (2021) in the context of cellwise outlier detection or Tolosana-Delgado et al. (2019) in the context of machine learning. Nevertheless, standard PCA still makes use of all PLRs, but not all of them are necessarily relevant for the multivariate data structure. Important information on the underlying processes in the data could be contained in a few distinct pairs, consisting of certain key parts. Standard PCA falls short, however, in easily identifying those PLRs that are most important for the data structure. It would therefore be desirable to first identify such relevant logratios which can then be used for further analysis, or even for the construction of interpretable logratio coordinates.

There have been several attempts to design a method that carefully selects PLRs; see for instance Greenacre (2018, 2019), whose STEP procedure is based on the construction of relevant, interpretable PLRs that build up a logratio coordinate system. Our aim in this paper is not to build the whole coordinate system out of the selected PLRs, but instead to focus on those PLRs that capture most information in the data. We therefore still resort to the context of PCA for compositional data but propose a specific version of it, namely sparse PCA, to identify the most relevant logratios (parts). We propose using the sparse PCA procedure introduced in Erichson et al. (2020), namely sparse PCA via variable projection, as it provides an effective and stable algorithm which is able to deal with the high dimensionality of PLRs.

The manuscript is structured as follows: In Sect. 2, we revise PCA for CoDa expressed in PLRs and introduce the sparse PCA method to identify important PLRs. A simulation study is provided in Sect. 3, and two geochemical data sets are analyzed in Sect. 4. Here, we also present various graphical tools to aid practitioners in their model interpretation. The final Sect. 5 ends with some concluding remarks.

## Sparse PCA with Pairwise Logratios

We start by revising principal component analysis (PCA) for CoDa expressed in PLRs in Sect. 2.1. In Sect. 2.2 we explain how one can use sparse PCA to identify important PLRs from the total candidate set.

### Principal Component Analysis with CoDa

Principal component analysis (PCA) is a well-known dimension reduction method for multivariate data, which is also commonly used as exploratory data analysis tools with CoDa. The key idea of PCA is to reduce the dimensionality of the original data set by constructing a new set of latent variables, called principal components (PCs), that are linear combinations of the original variables and mutually uncorrelated. PCs are constructed such that the first PC maximizes the variance in the original data, and subsequent PCs maximize the remaining variance. For CoDa, all computations need to be done in orthonormal coordinates, and we refer to Filzmoser et al. (2018) for further details on PCA with CoDa.

Let ${\textbf{X}}=(x_{ij})_{1\le i \le n, 1\le j \le D}$ be a matrix carrying information about a *D*-part composition (in the columns) with *n* observations (in the rows). We represent the CoDa through a matrix of PLRs with $\frac{D(D-1)}{2}$ columns (as the information carried in $\textrm{ln}\frac{x_{i}}{x_{j}}$ is the same as in $\textrm{ln}\frac{x_{j}}{x_{i}}$), which will be further denoted as ${\textbf{X}}_\textrm{pair}$. It is assumed that the matrix ${\textbf{X}}_\textrm{pair}$ is centered prior to performing PCA. Note that centering of the input matrix prior to PCA is a standard procedure. Having a centered matrix, we then account for the real variance (not affected by the shift caused by non-centered data).

To construct the PCs, we adopt the notation of Erichson et al. (2020), to be further used in Sect. 2.2. The *i*th $\left( i=1,\ldots , \frac{D(D-1)}{2}\right)$ principal component score ${\textbf{z}}_{i}$ is the *n*-dimensional (column) vector formed as a linear weighted combination of all PLRs

1 $$\begin{aligned} {\textbf{z}}_{i} = {\textbf{X}}_\textrm{pair} {\textbf{p}}_{i}, \end{aligned}$$

where ${\textbf{p}}_{i}$ is a $\frac{D(D-1)}{2}$-dimensional (column) vector of loadings. All PCs ${\textbf{z}}_{1}, \ldots , {\textbf{z}}_{\frac{D(D-1)}{2}}$ can then be combined in an $n\times \frac{D(D-1)}{2}$ score matrix ${\textbf{Z}}$ given by

2 $$\begin{aligned} {\textbf{Z}} = {\textbf{X}}_\textrm{pair} {\textbf{P}}. \end{aligned}$$

Note that, apart from the fact that only up to $D-1$ PCs have nonzero variance, one typically does not consider all but instead only the first few to capture a desirable fraction of the total variability in the original data.

After having obtained the PCA model, one can proceed to visualization in a compositional biplot, that is, a graphical display of both variables (loadings) and observations (scores). As such, one can assess the importance of each PLR, group of logratios, or observation for explaining the total variability; see for instance Daunis-i Estadella et al. (2011).

However, as the dimensionality of the composition increases, it becomes cumbersome to identify those logratios or even key original components that are most relevant for revealing leading processes in the data. This calls for a regularization technique that can identify such important logratios. To this end, we resort to sparse PCA, as discussed in the next section.

### Selecting Important Pairwise Logratios via Sparse PCA

We use the sparse PCA method, based on variable projection, of Erichson et al. (2020) to encourage sparsity in the PCA loadings of all $\frac{D(D-1)}{2}$ PLRs. This method is based directly on the input data matrix (instead of a covariance matrix), and it can cope with high-dimensional data having more variables than observations. The method aims to find a set of sparse loading vectors, that is, vectors with just a few nonzero elements, by minimizing the objective function

3 $$\begin{aligned} \min _{{\textbf{H}},{\textbf{B}}} \ \frac{1}{2} \Vert {\textbf{X}} - {\textbf{X}}{\textbf{B}}{\textbf{H}}^T \Vert _{F}^{2} + \psi ({\textbf{B}}),\quad \mathrm{{subject\,to}}\quad {\textbf{H}}^T{\textbf{H}} = {\textbf{I}}, \end{aligned}$$

where ${\textbf{X}}$ is the data matrix (to be clarified below for the CoDa case), ${\textbf{B}}$ is a sparse loading (weight) matrix, ${\textbf{H}}$ is an orthonormal matrix, $\psi$ denotes a sparsity-inducing penalty function, and $\Vert \cdot \Vert _{F}$ stands for the Frobenius norm ($\Vert {\textbf{A}} \Vert _{F} = \sqrt{\text {trace}({\textbf{A}}^{T}{\textbf{A}})}$). For the penalty function, we take an elastic net penalty as given by

4 $$\begin{aligned} \psi ({\textbf{B}}) = \alpha \Vert {\textbf{B}}\Vert _{1} + \beta \Vert {\textbf{B}} \Vert _{2} ^2,\quad \end{aligned}$$

where $\alpha , \beta >0$ are tuning parameters. The elastic net penalty was introduced in Zou and Hastie (2005) as a combination of an $\ell _1$ penalty (lasso, Tibshirani, 1996) and an $\ell _2$ penalty (ridge, Hoerl and Kennard, 1970). The tuning parameter $\alpha$ controls the degree of sparsity of the resulting loading vectors: the higher its value, the sparser the result. The $\ell _1$ penalty and its corresponding tuning parameter $\alpha$ share their variable selection property with the lasso, and it is therefore this part of the penalty that offers the interpretability of sparse PCA. The tuning parameter $\beta$ results in shrinkage of the parameters towards zero (no exact zeros, so no variable selection), but does help to handle collinearity, just as the ridge does, without hindering the interpretability advantage obtained through the $\ell _1$ penalty. For the tuning parameter $\beta$, we take the default setting of $\beta =0.0001$ as used in Erichson et al. (2020). (One can use a simple lasso penalty instead of an elastic net penalty by setting $\beta = 0$. We verified that results similar to those reported in the paper are obtained in such a case.) The resulting estimated PCs are then defined as ${\textbf{Z}} = {\textbf{X}}\widehat{{\textbf{B}}}$. While standard PCA is orthogonally equivariant, meaning that any olr coordinates or a set of all $\frac{D(D-1)}{2}$ PLRs would lead to the same scores, this is no longer the case with sparse PCA because of the $\ell _1$-regularization. Using all $\frac{D(D-1)}{2}$ PLRs as seminal information in CoDa for PCA is therefore not only a natural choice with respect to the aim of our analysis, but also an essential requirement for obtaining consistent results.

The procedure to perform sparse PCA for CoDa expressed in PLRs can then be summarized into the following two simple steps:

1. Construct a matrix of all $\frac{D(D-1)}{2}$ PLRs, and mean-center this matrix, denoted as ${\textbf{X}}_\textrm{pair}$.
2. Perform sparse PCA by minimizing the objective function (3) with the matrix ${\textbf{X}}_\textrm{pair}$, and let $\widehat{{\textbf{B}}}$ denote the estimated loadings matrix.

The sparse PCA procedure thus results in a sparse loading matrix $\widehat{{\textbf{B}}}$ of dimension $\frac{D(D-1)}{2} \times \frac{D(D-1)}{2}$. The importance of each PLR can then be studied from the sparsity pattern in the corresponding row of the loadings matrix.

In the remainder of this paper, we focus only on the first two PCs, as they retain the most important variability. The loadings matrix then has only two columns to be inspected, and the scores matrix also consists of two columns. This information is typically presented in a biplot, which allows us to study the relationships between loadings and scores (Aitchison and Greenacre 2002).

Moreover, we further talk about logratios with zero loadings (in short, zero logratios) as those logratios having zero loadings in both the first and second PC. It is important to point out that one does not have to limit the analysis to the first two PCs. In fact, one can choose *k* PCs, $k = 1,\dots ,\frac{D(D-1)}{2}$, but as stated above, it should be noted that only up to $D-1$ PCs have nonzero variance.

All calculations were performed in R (R Core Team 2023). To perform sparse PCA as described above, the function spca available in the package sparsepca (Erichson et al. 2018) was used. The code for our algorithm is available on the GitHub page of the first author (https://github.com/NesrstovaV/PairwiseLogrs-sPCA.git).

## Simulation Study

We perform a simulation study to investigate the ability of the proposed method to identify important PLRs among the total set. In Sect. 3.1, we describe the simulation scenarios. In Sect. 3.2 we compare our sparse PCA proposal against the STEP method of Greenacre (2019) before further zooming in to the performance of sparse PCA in Sect. 3.3.

### Simulation Scenarios

We consider a data generation process via balance coordinates consisting of a combination of “relevant” and “noise” balances. As such, we embed “sparsity” in the compositional parts, since the compositional parts in the relevant coordinates are considered important, whereas parts in the noise coordinates are unimportant and hence should display zero loadings in the sparse PCA procedure.

We consider three simulation scenarios, each of them in combination with either $$D=10$$ parts or $$D=20$$ parts. Table 1 summarizes the three simulation scenarios with an SBP scheme for a $$D=10$$-part composition with nine balances. An SBP is usually encoded in a sign matrix, which illustrates the division of parts into groups as can be seen from Table 1. Typically, parts in the numerator are coded as “$$+$$” and parts in the denominator as “−.” Parts that are not included in a particular balance are coded as “0.” The scenarios in Table 1 differ in terms of the proportion of relevant versus noise balances. In scenario A, there is a (roughly) equal amount of relevant ($$b_1-b_5$$) and noise ($$b_6-b_9$$) balances in the coordinate representation. In contrast, in scenario B the relevant balances ($$b_1-b_7)$$ dominate, while in scenario C the noise balances ($$b_3-b_9)$$ dominate.

**Table 1:**
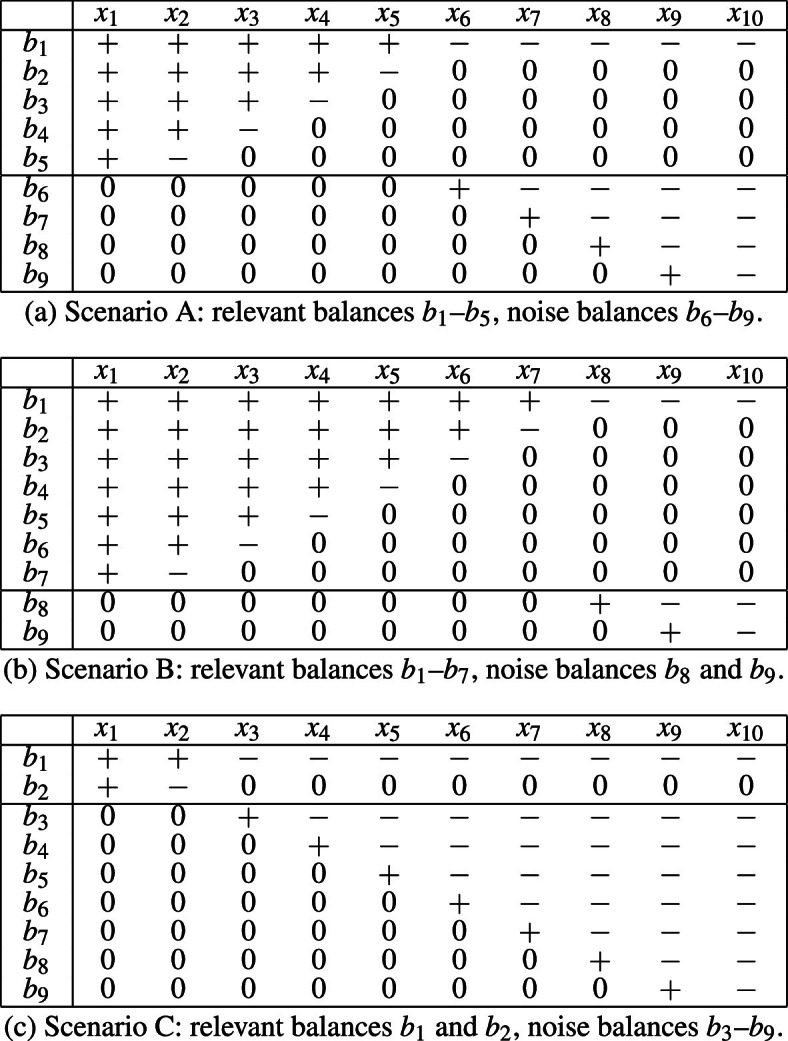
SBP tables of the different scenarios for $D=10$

Cells with the “$+$” (“−”) sign mark parts in the numerator (denominator) of the balance coordinate. Zeros mark parts not included in the balance

In addition to a $D=10$-part composition, we also consider $D=20$ with 10 (scenario A), 15 (scenario B), and four (scenario C) relevant balances; the SBP was performed analogously to the case for $D=10$. While these compositions appear to be of moderate size at first sight, the number of PLRs in each is considerable, namely 90 and 380, respectively (or 45 and 190, respectively, when logratios are considered up to sign).

The relevant and noise balance coordinates are generated such that their variability reflects settings that typically occur in CoDa data sets. Specifically, the relevant balances are drawn from a multivariate normal distribution with mean zero and covariance matrix with 1s on the diagonal and 0.7 for the off-diagonal elements. The noise balances are drawn from a uniform distribution $U(-2,2)$. We then back-transform the system of all balance coordinates to obtain an $n\times D$ data matrix ${\textbf{X}}$ of raw compositions. We fix the number of observations to $n=100$ and use 100 simulation runs.

### Comparing Sparse PCA to STEP

We start by comparing the proposed sparse PCA approach to the method reported by Greenacre (2019). The latter is called stepwise ratio selection (STEP) and is available in the function STEP of the R package easyCODA (Greenacre 2020). STEP is a well-established method to search for a set of important PLRs that serves as a suitable representation of the whole compositional data set. Moreover, this method is also suitable for dimension reduction (as is PCA), apart from other methods focused on the choice of PLRs, such as the work of Coenders and Greenacre (2023), where problems related to the regression context are discussed. As STEP constructs logratio coordinates, it chooses at maximum $D-1$ PLRs (i.e., coordinates), such that these logratios are mutually non-collinear. Therefore, we compare our proposal to STEP through $D-1$ PLRs, the maximum we can obtain with the STEP algorithm. STEP orders the logratios according to their percentage of explained variance, with the first one having the highest percentage of explained variability. Moreover, the logratios are chosen such that they are not linearly dependent on each other. For example, if logratios $\textrm{ln}\frac{x_{i}}{x_{j}}$ and $\textrm{ln}\frac{x_{j}}{x_{k}}$ are chosen, then the logratio $\textrm{ln}\frac{x_{i}}{x_{k}}$ can no longer be selected.

**Fig. 1:**
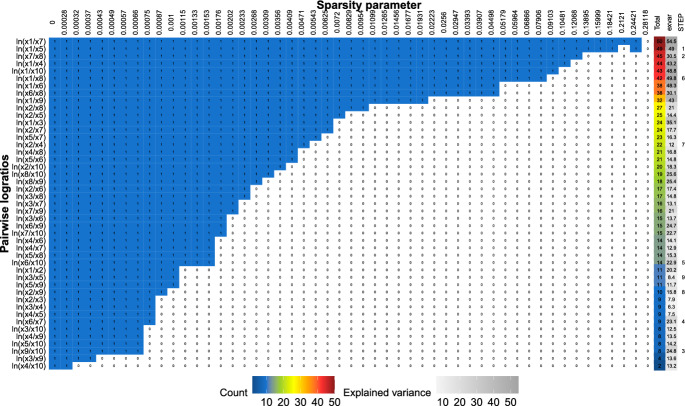
Stability plot for an illustrative example of simulation scenario A with $D=10$. The graph shows stability paths for PLRs (in rows) where colored cells with 1s reflect selection of the PLR (i.e., nonzero loading) for the sparse PCA solution with particular $\alpha$ (in columns, until full sparsity is reached). The column “total” displays the number of models (row counts) out of 51 in which the PLR is selected; the column “exvar” gives the explained variability by the logratio. The last column “STEP” shows the ranks of $D-1$ PLRs selected by STEP

To select $D-1$ logratios for our proposal, we first sort (in each simulation run) the PLRs according to their stability, and then keep only the first $D-1$ PLRs for comparison with STEP. A logratio is considered to be more stable than another if it remains selected for higher values of the sparsity parameter $\alpha$. We apply the sparse PCA method for different values of $\alpha$, ranging from the solution with no sparsity ($\alpha =0$) to maximal sparsity ($\alpha _{\text {max}}$, which varies from setting to setting). We consider a logarithmically spaced grid of 51 sparsity parameters between these extreme values and apply the sparse PCA procedure for each value in the grid. We then display stability paths (see, e.g., Fig. 1), similar to regularization paths in the context of sparse regression analysis.

Figure 1 presents an illustrative example (namely for one simulation run for scenario A, $D = 10$) of the stability of each logratio for increasing values of the sparsity parameter $\alpha$ on the horizontal axis. On the vertical axis we show the $\frac{D(D-1)}{2}$ PLRs. (We plot only $\frac{D(D-1)}{2}$ PLRs to avoid redundancy. For example, logratios $\textrm{ln}\frac{x_{1}}{x_{5}}$ and $\textrm{ln}\frac{x_{5}}{x_{1}}$ differ just by sign, and the percentage of variability explained by each is therefore the same.) The cells then highlight the selection results: 1s in blue cells correspond to selected logratios (i.e., with nonzero loading); zeros in white cells correspond to nonselected logratios (i.e., with zero loading). We order the PLRs according to the number of times they are selected across all the grid points of the sparsity parameter (see the Total column in Fig. 1). The most stable logratios thus appear at the top and remain nonzero even in very sparse models, as can be seen from the longer blue stretches. The column “exvar” shows the variability explained by each PLR. Finally, the last column “STEP” shows the ranks of the $D-1$ PLRs as selected by the STEP algorithm.

Once we have a set of $D-1$ selected PLRs for our proposal as well as for STEP, we can then investigate their performance. We compare our sparse PCA proposal to STEP on two criteria, namely (i) the percentage of important PLRs chosen by each method, and (ii) the rank of the chosen logratios based on their variances.

#### Selection of Important Logratios

**Fig. 2:**
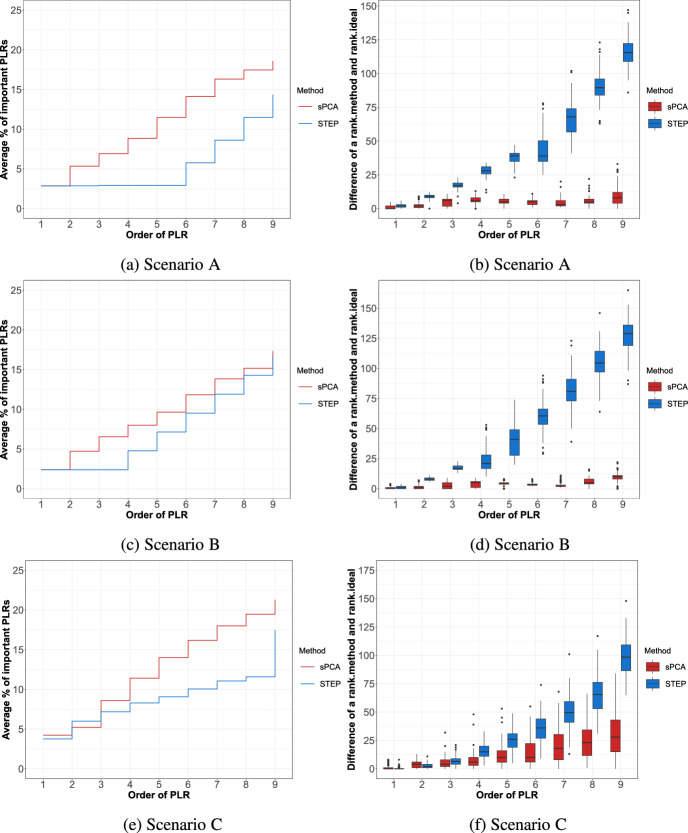
Comparison of sparse PCA versus STEP on simulation scenarios **A**, **B**, and **C** for $D=10$. Left: average percentage of correctly identified important PLRs for sPCA (red) and STEP (blue) for the first $D-1$ ordered PLRs (PLR) on the horizontal axis. Right: boxplots showing the differences between the cumulative ranks of the PLR obtained from sPCA (red) or STEP (blue) and the ideal ranks

Given the set of $D-1$ PLRs obtained from sparse PCA (i.e., the most stable ones) and STEP, we can investigate whether these belong to the set of important PLRs (corresponding to the relevant balances). In the left panels of Fig. 2, we provide step plots that display the average percentage, across simulation runs, of important PLRs that are picked up in the first $D-1$ selected PLRs of sparse PCA (sPCA, in red) and STEP (in blue). We display the results on the different simulation scenarios for $D=10$ in the respective panels of Fig. 2a, c, and e. Results for all scenarios of $D = 20$ are reported and summarized in Appendix A, as these are very similar.

Sparse PCA is, overall, more successful in capturing the important PLRs than STEP, since the increase in percentage of correctly captured important logratios occurs faster than for STEP. The discrepancy between the two is the largest for scenario A. In scenario C, where the data contain few important PLRs, both methods perform similarly for the first two chosen logratios, but further on, sPCA outperforms STEP again. Importantly, sparse PCA, overall, does not perform worse than STEP. It is therefore a suitable alternative for choosing important PLRs if a complete coordinate system formed by PLRs is not a primary goal, even more so since STEP is limited by the number of chosen logratios ($D-1$) whereas our proposal is not.

#### Ranking Based on Variances

Next, we compare sparse PCA and STEP based on the ranks of the selected PLRs. We start by computing the “ideal” rank of each PLR, thereby ranking them according to their variances, with the logratio having the highest variance being ranked first. For sparse PCA, we obtain the first $D-1$ logratios ordered according to stability; for STEP we simply obtain the $D-1$ ordered logratios. For each method, we then compute the cumulative ranks for the selected logratios, going from 1 up to $D-1$. In the case of sPCA, we first assign ranks to all $\frac{D(D-1)}{2}$ logratios (ordered by stability) and then keep only the first $D-1$ most stable ones. Finally, we obtain the difference between these cumulative sum of ranks and the ideal cumulative sum of rank. By definition, this difference can only be positive. If the difference is zero, the logratios ordered by the method exactly correspond to logratios ranked by their variance. The larger the difference, the higher the tendency of the method to rank logratios with lower variances before logratios with higher ones.

Boxplots in the right panel of Fig. 2 show the variability of these differences across the simulation runs, and this for the different simulation scenarios. Sparse PCA outperforms STEP considerably, as the difference with the ideal ranks is smaller and the margin by which sparse PCA outperforms STEP gradually increases as more PLRs are considered. This implies that logratios with high variance typically appear as the most stable ones in our sparse PCA procedure, which is a desirable feature.Given the good performance of our sparse PCA proposal against STEP, we further zoom in to its performance across the different simulation scenarios in the next section.

### Performance of Sparse PCA Across the Simulation Scenarios

We discuss the performance of sparse PCA in terms of (i) the trade-off of explained variability with model parsimony and (ii) the ability to correctly separate the important logratios (corresponding to the relevant balances) from the unimportant logratios (corresponding to the noise balances). To this end, we compute the false positive rate (FPR) and false negative rate (FNR) (Fawcett 2006) given by

$$\begin{aligned} \textrm{FPR} = \frac{\text {FP}}{{\text {FP}} + {\text {TN}}} \qquad {\text {FNR}} = \frac{\text {FN}}{{\text {FN}}+{\text {TP}}}, \end{aligned}$$

where the true positives TP (true negative TN) indicates the number of true important, nonzero (unimportant, zero) logratios. The false positives FP (false negatives FN) then give the number of truly unimportant, zero (important nonzero) logratios that are incorrectly given a nonzero (zero) loading.

We are now ready to discuss the results for each of the three simulation scenarios with $D=10$. For $D=20$, the main findings remain unchanged; we therefore report and summarize these results in Appendix A.

**Fig. 3:**
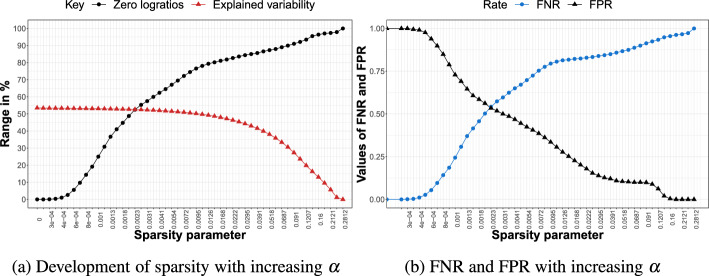
Simulation scenario A for $D=10$. Left: percentage of zero logratios (black) and explained variability (red) for different values of the sparsity parameter $\alpha$. Right: FPR (black) and FNR (blue) for different values of $\alpha$

#### Results Scenario A

Figure 3 shows the results for simulation scenario A with $D = 10$ parts. Figure 3a displays the percentage of explained variability (in red) and percentage of zero logratios (in black) across the different values of the sparsity parameter $\alpha$. Note that for $\alpha =0$, the percentage of explained variability does not start at 100%, since we focus only on the first two PCs. When gradually increasing the sparsity parameter, we see that the percentage of explained variability only mildly decreases, whereas the percentage of zero logratios increases much more quickly. This trade-off provides interesting opportunities to consider sparser solutions with (few) important logratios. Indeed, the dense, hard-to-interpret solution ($\alpha =0$) offers the highest attainable percentage of explained variability (53.5%), but a considerably sparser solution with up to 52.6% zero logratios (around $\alpha = 0.0023$) only pays a price in terms of explained variability of around 0.9% (i.e., drop from 53.5% to 52.6% in explained variability). The PCA solution with that same sparsity parameter attains the best possible balance between the FNR and FPR, which are both roughly at 0.5, as can be seen from Fig. 3b. Note that, by definition, the FPR decreases from 1 to 0 as the degree of sparsity increases from zero to maximal sparsity, while the opposite occurs for the FNR.

**Fig. 4:**
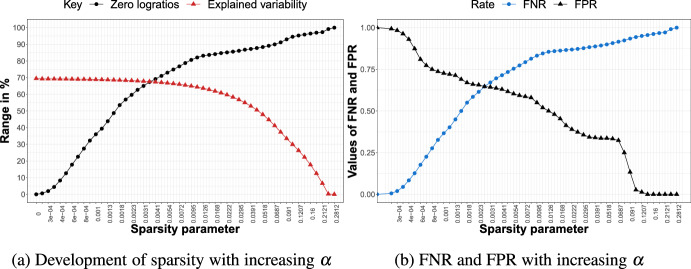
Simulation scenario B for $D=10$. Left: percentage of zero logratios (black) and explained variability (red) for different values of the sparsity parameter $\alpha$. Right: FPR (black) and FNR (blue) for different values of $\alpha$

#### Results Scenario B

Results for scenario B are shown in Fig. 4. Having more relevant balances leads to an overall higher percentage of explained variability (roughly 70%) for the dense solution. Similarly to scenario A, the percentage of explained variability decreases slowly for sparser solutions, but the sparsity of the loadings shoots up more quickly. For a sparse solution where roughly half of the logratios have zero loadings, still almost 69% of the variability can be explained. In this model, the $FNR=0.5$, which is considerably below the $FPR (0.69)$, meaning that fewer important logratios are missed (impacting the FNR) than unimportant logratios are selected (impacting the FPR).

**Fig. 5:**
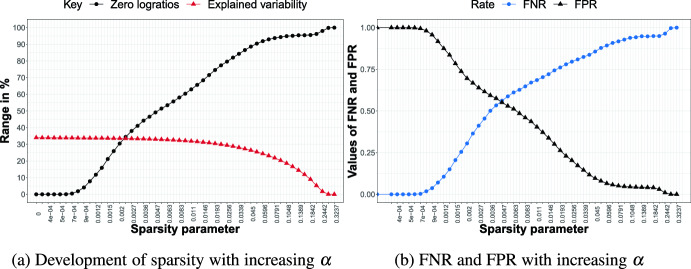
Simulation scenario C for $D=10$. Left: percentage of zero logratios (black) and explained variability (red) for different values of the sparsity parameter $\alpha$. Right: FPR (black) and FNR (blue) for different values of $\alpha$

#### Results Scenario C

Figure 5 shows the results for the last scenario having the most noise balances. The maximum percentage of explained variability (at the dense model) now drops below 40% but we still see only a moderate decline in explained variability combined with a steeper rise in sparsity when the sparsity parameter $\alpha$ gradually moves away from zero. Finally, the rate of increase in FNR, tracking the omission of important logratios, is slower in scenario C (Fig. 5b) than in scenario B (Fig. 4b). Hence, when there are only a few relevant balances, the important logratios survive (i.e., display nonzero loadings) for a longer range of increasing values of the sparsity parameter.

**Fig. 6:**
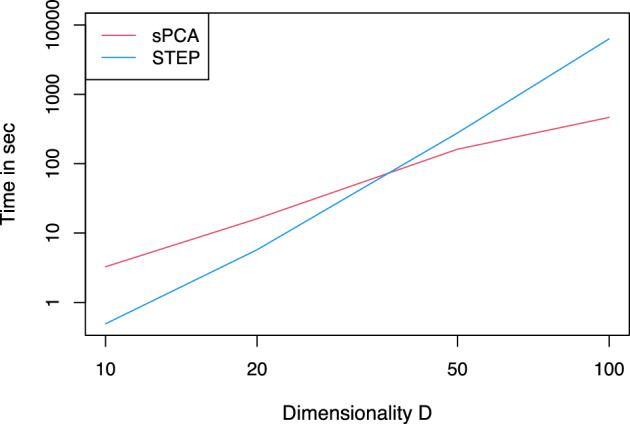
Comparison of computation time (average values over 20 runs; shown on a log–log scale) of sPCA (red) and STEP (blue) for scenario B and various choices of dimension *D*

Finally, we provide a comparison of sPCA and STEP in terms of computation time. The calculations were performed on a Windows laptop (Intel Core i7-4810MQ, four cores, eight logical processors, frequency 2.80GHz, RAM 16 GB). We measured computation times for all three scenarios and different dimensions of the data, $D = \{10, 20, 50, 100\}$. The comparison between STEP and sPCA is visualized in Fig. 6, where the average of computation time over 20 simulation runs is shown for each dimension on a log–log scale. In general, computing times for all three scenarios show the same tendency; thus, for brevity, we show results for scenario B only. For data sets of smaller dimensions (i.e., $D=10$ or 20 parts), sPCA and STEP perform similarly and are quite fast, with STEP slightly outperforming sPCA in these two low-dimensional settings. On the other hand, starting with $D = 50$, STEP starts to be computationally more expensive than sPCA. This is clearly seen for the high-dimensional case having $D = 100$, where the computation time for STEP rises sharply.

## Applications

In this section, we demonstrate the usefulness of the sparse PCA proposal on two empirical data sets in identifying potentially relevant PLRs (and thus important parts of compositions). Both data sets consist of geochemical elements. The first data set contains compositions of glacial sediments; the second data set comes from archaeometry.

### Aar Massif Data

The Aar data set, introduced in von Eynatten et al. (2012), contains geochemical CoDa of glacial sediments from the Aar Massif in Switzerland, specifically the Rhone, Damma, and Sidelen glaciers. The data are publicly available in the R package compositions (van den Boogaart et al. 2021). It consists of two groups of components: major oxides and trace elements. However, as the amount of trace elements is negligible compared to major oxides, we focus only on the latter. We analyze a composition consisting of $D=10$ oxide components, namely $\textrm{SiO}_{2}$, $\textrm{Al}_{2}\textrm{O}_{3}$, $\textrm{TiO}_{2}$, $\textrm{Fe}_{2}\textrm{O}_{3}^{tot}$ (total iron(III) oxide, further noted as 3*t*), MnO, MgO, CaO, $\textrm{Na}_{2}\textrm{O}$, $\textrm{K}_{2}\textrm{O}$, and $\textrm{P}_{2}\textrm{O}_{5}$, all expressed in weight percent. There are $n=87$ observations (i.e., samples) available on this 10-part composition. Hence, the total number of PLRs is $D(D-1) = 90$. Finally, there are no zero values in the data; hence imputation of zeros is not necessary. Similarly to Sect. 3, we use sparse PCA to compute the first two PCs for (51) different values of the sparsity parameter $\alpha$, ranging from zero to the value $\alpha _{\text {max}}$ resulting in full sparsity.

**Fig. 7:**
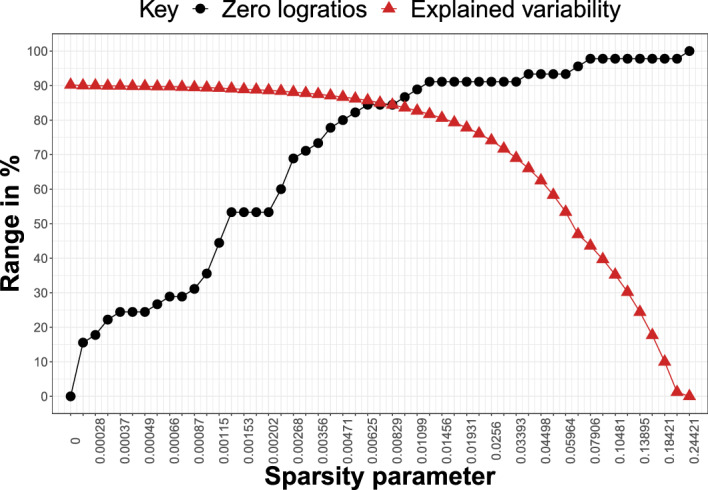
Aar Massif data: percentage of zero logratios (black) and explained variability (red) by the first two PCs in the sparse PCA model with sparsity parameter $\alpha$ (on horizontal axis)

Figure 7 shows the trade-off between sparsity and explained variability for different values of the sparsity parameter $\alpha$. The percentage of explained variability in the dense model is high, around 90%. This percentage declines only slowly and remains around 88% even when more than half of the PLRs are set to zero (from $\alpha = 0.0013$ to $\alpha = 0.002$). Moreover, when the sparsity level is fairly high (almost 85% of zero logratios, $\alpha = 0.0063$), the percentage of explained variability is still around 86%. Sparse PCA solutions in this neighborhood still offer high percentages of explained variability (note that the percentage of explained variability drops below 80% for models where slightly more than 90% of PLRs were set to zero) and are thus good candidate solutions to identify important logratios. In addition, from the simulation study, we know that the best balance in terms of FPR and FNR is typically reached for values of the sparsity parameter where the two lines (of explained variability and zero loadings) cross. This happens when there are more than 84% of zero PLRs and $\alpha = 0.0072$ (or the two neighboring values of $\alpha$, as for each we reach the same sparsity, and the explained variability changes just slightly from 85.7 to 84.4%). Finally, note that the PCA solutions with the highest sparsity levels (i.e., solutions corresponding to the largest $\alpha$ values) should be omitted from one’s consideration, as the (close to maximal) sparsity level makes the model meaningless due to the zero percentage of explained variability.

**Fig. 8:**
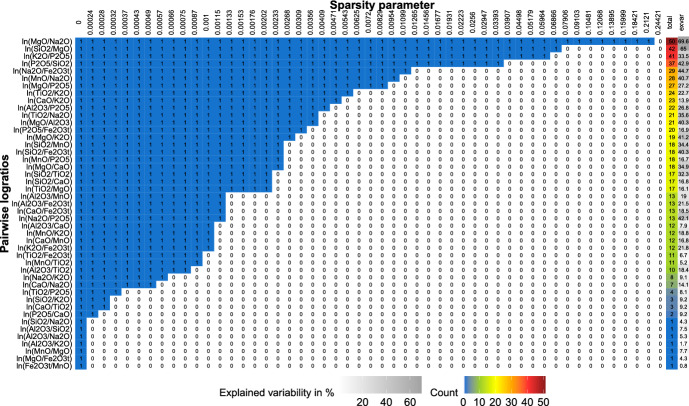
Aar Massif data: stability paths for PLRs (in rows) where colored cells with 1s reflect selection of the PLR (i.e., nonzero loading) for the sparse PCA solution with particular $\alpha$ (in columns). The column “total” displays the number of models (row counts) out of 51 in which the PLR is selected, the column “exvar” gives the percentage of variability explained by the logratio

Next, we investigate the stability selection of each PLR across the sparse PCA models (with varying sparsity parameters) through the stability paths depicted in Fig. 8, which is similar to Fig. 1 in the simulation study. From Fig. 8, we observe that the logratios ${\text {ln}}\frac{\text{ MgO }}{{\text{ Na }}_{2}{\text{ O }}}$, ${\text {ln}}\frac{{\text{ SiO }}_{2}}{\text{ MgO }}$, ${\text {ln}}\frac{{\text{ K }}_{2}{\text{ O }}}{{\text{ P }}_{2}{\text{ O }}_{5}}$, and ${\text {ln}}\frac{{\text{ P }}_{2}{\text{ O }}_{5}}{{\text{ SiO }}_{2}}$ are very stable, as they are selected across more than 35 considered models (with ${\text {ln}}\frac{{\text{ MgO }}}{{\text{ Na }}_{2}{\text{ O }}}$ being the most stable one), which can be seen from the row counts in the second-to-last column of the figure. Hence, these PLRs are to be considered important. In the last column of Fig. 8, we display the contribution of each PLR to the total explained variability. We see that the most stable PLRs also display among the largest contributions to the explained variability, ranging from almost 70% to roughly 34%. At the other end of the spectrum, our sparse PCA procedure returns zero loadings for altogether seven PLRs across practically all models except the dense one, thereby deeming these rather unimportant. However, these logratios differ in the explained variability, ranging from around 7.5% for ${\text {ln}}\frac{\textrm{Al}_{2}\textrm{O}_{3}}{\textrm{SiO}_{2}}$ to roughly 0.8% for ${\text {ln}}\frac{\textrm{Fe}_{2}O_{3\textrm{t}}}{\textrm{MnO}}$.

von Eynatten et al. (2012) analyzed the Aar Massif data via PCA using a centered logratio (clr) representation of the data (note that clr is considered a typical representation for CoDa in the context of PCA; see Aitchison and Greenacre, 2002). The clr coefficients are defined as $\textrm{clr}({\textbf{x}}) = \left( \textrm{log}\frac{x_{1}}{g({\textbf{x}})},\dots ,\textrm{log}\frac{x_{D}}{g({\textbf{x}})}\right)$, where $g({\textbf{x}})$ stands for the geometric mean of the parts (Pawlowsky-Glahn et al. 2015). The resulting biplot is depicted in Fig. 10, and matches the biplot shown in von Eynatten et al. (2012) up to a rotation. The links between the arrows (representing the compositional parts) indicate the variability in a pairwise logratio of these particular parts: the larger the distance between the two links, the larger (approximately) the variability in a logratio consisting of these parts. In the first PC (horizontal axis), the dominant elements with positive values were Si, Na, Al, and K, while Mg, Fe, Mn, Ti, and P earned negative score values. Moreover, the oxides with Si, Al, Na, and K (referred to as felsic elements) stand opposite to Mg, Fe and Mn (mafic elements). Hence, the (linear) decrease in felsic elements with respect to mafic elements can characterize the trend of samples going from coarse sand to clay (von Eynatten et al. 2012). Looking at Fig. 8, it can be seen that the first most stable logratios $\textrm{ln}\frac{\textrm{MgO}}{\textrm{Na}_{2}\textrm{O}}$ and $\textrm{ln}\frac{\textrm{SiO}_{2}}{\textrm{MgO}}$ (also accounting for the highest percentage of explained variability) are both a combination of felsic-mafic elements. Now, relying solely on the biplot in Fig. 10, the identification of pairwise logratios with the highest explained variability is, however, quite challenging. In contrast, the presented sparse PCA complemented with a visualization via a heatmap, as in Fig. 8, offers a more straightforward solution for identifying relevant pairwise logratios.

**Fig. 9:**
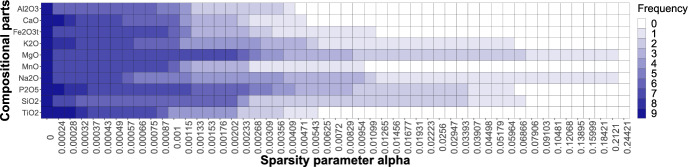
Aar Massif data: heatmap of compositional parts (in rows) for the sparse PCA solution with particular $\alpha$ (in columns). The darker the shading, the higher the occurrence of the part across nonzero PLRs

Next, we further zoom out to the compositional parts and compute their frequency of occurrence and stability across the sparse PCA models. In Fig. 9, we now display the 10 components on the vertical axis, while keeping the sparsity parameter on the horizontal axis. Each cell then displays the number of nonzero PLRs in which the particular part appears. The darker the cell, the more frequently the part occurs; in addition, the further the colored shading stretches to the right of the plot, the higher the stability of the part. We observe that MgO, $\textrm{P}_{2}\textrm{O}_{5}$, and $\textrm{SiO}_{2}$ are most frequently selected in the first 17 or 18 models. From another perspective, MgO, $\textrm{Na}_{2}\textrm{O}$ (followed by $\textrm{K}_{2}\textrm{O}$, $\textrm{P}_{2}\textrm{O}_{5}$, and $\textrm{SiO}_{2}$) are most stable, as they are present in the majority of models, even though not as frequently. This is not surprising, as the PLRs with MgO, $\textrm{Na}_{2}\textrm{O}$, ${\text {K}}_{2}{\text {O}}$, ${\text {P}}_{2}{\text {O}}_{5}$, and ${\text {SiO}}_{2}$ turned out to be within the most stable ones (see Fig. 8). In addition, the elements Mg, Na, Si, and P are documented to have the largest contribution to the explained variability in von Eynatten et al. (2012). We also find these to play a prominent role in the heatmap of Fig. 9, most of them mainly in terms of stability, but Mg, P, and Si (in parts MgO, $P_2O_5$, and ${\text {SiO}}_{2}$) also in terms of frequency of occurrence.

**Fig. 10:**
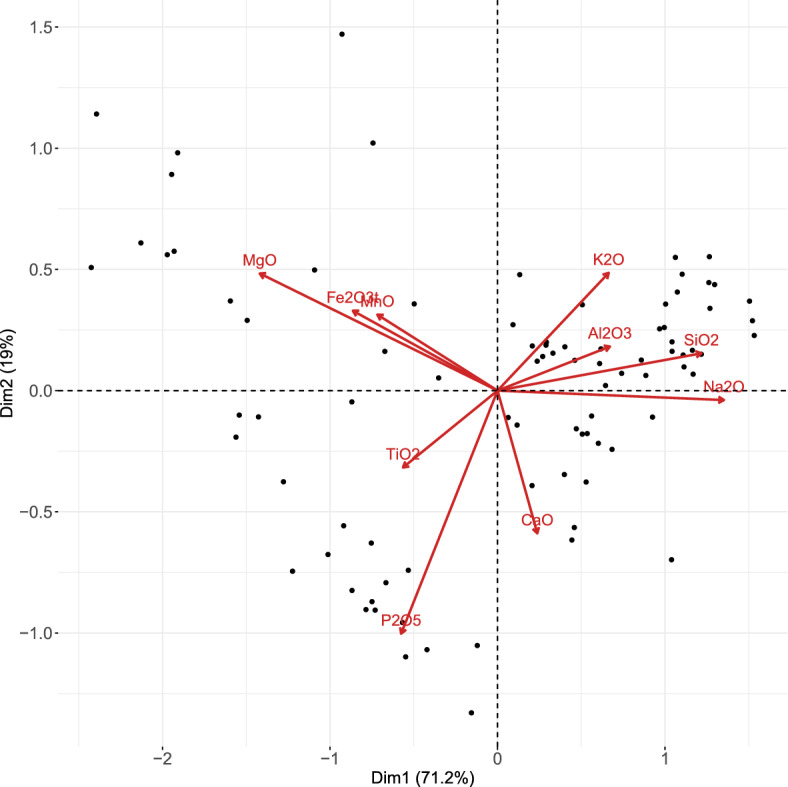
Aar Massif data: PCA biplot of the clr representation of the data

### Archaeometric Data

Our second data set contains samples of Roman colorless cylindrical glass cups found in an archaeological site in eastern England (Colchester) and is originally a subject of study of the Romano-British Glass Project at Leeds University and the Ancient Monuments Laboratory, English Heritage. The data set is introduced in Baxter et al. (1990) and also analyzed in Greenacre (2019). It is publicly available in the R package easyCODA (Greenacre 2020). We have a $D=11$-part composition (trace elements were omitted) of elements silicon (Si), aluminum (Al), iron (Fe), magnesium (Mg), calcium (Ca), sodium (Na), potassium (K), titanium (Ti), phosphorus (P), manganese (Mn), and antimony (Sb) for which $n=47$ observations are available. There are no zeros in the data; hence imputation is not needed.

**Fig. 11:**
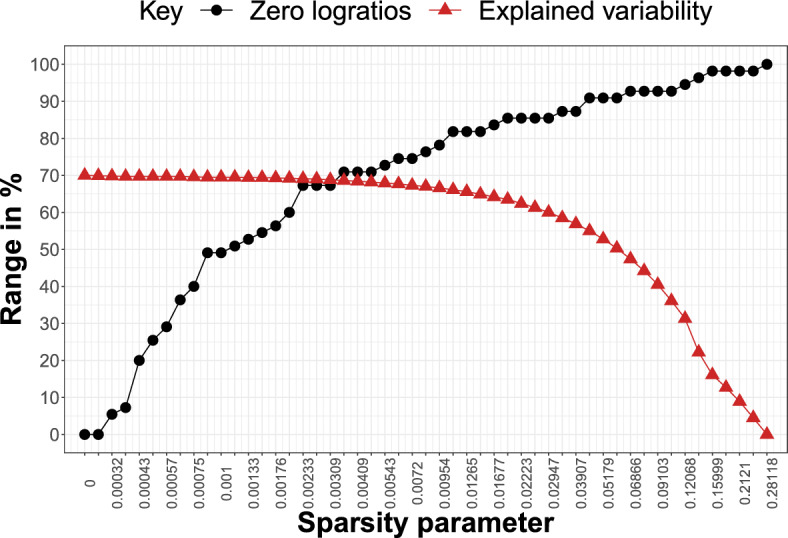
Archaeometric data: percentage of zero logratios (black) and explained variability (red) by the first two PCs in the sparse PCA model with sparsity parameter $\alpha$ (on horizontal axis)

We conduct the same analysis as for the Aar Massif data set. Figure 11 gives the trade-off between sparsity and explained variability across the various sparse PCA solutions. It can be observed that sparsity for $\alpha = 0.001$ results in a model with roughly 50% of zero PLRs while still reaching almost 70% of explained variability. Interestingly, the percentages of explained variability remains very high and, importantly, flat at the start of the plot. For more than 60% zero PLRs, the explained variability remains almost 70%. This data set therefore seems to lend itself well to sparsification.

**Fig. 12:**
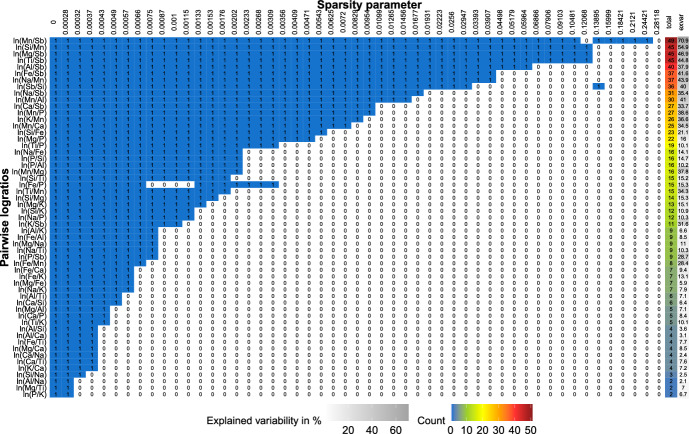
Archaeometric data: stability paths for PLRs (in rows) where colored cells with 1s reflect the selection of the PLR (i.e., nonzero loading) for the sparse PCA solution with particular $\alpha$ (in columns). The column “total” displays the number of models (row counts) out of 51 in which the PLR is selected; the column “exvar” gives the percentage of variability explained by the logratio

Next, we inspect the stability of the PLRs in Fig. 12. The most stable PLRs are $\textrm{ln}\frac{\textrm{Mn}}{\textrm{Sb}}$, $\textrm{ln}\frac{\textrm{Si}}{\textrm{Mn}}$, $\textrm{ln}\frac{\textrm{Mg}}{\textrm{Sb}}$, and $\textrm{ln}\frac{\textrm{Ti}}{\textrm{Sb}}$, with explained variability of 70.85%, 54.88%, $46.91\%$, and $44.83\%$, respectively. These logratios appeared to be nonzero in more than 45 models. One can observe a slightly odd gap for $\alpha = 0.12068$ considering the first logratio and three other cells which do not follow the assumption of continuously increasing sparsity. This could be the result of certain slight instability of the procedure in the presence of a complex data structure. Nevertheless, these effects are very minor. Apart from the aforementioned four most stable logratios, there is another group of four logratios having nonzero loadings across more than 35 (i.e., more than half, having altogether 51 models) sparse PCA solutions. When considering the sparsest model including only the four most stable logratios (for $\alpha =0.10481$, as in the next model the first logratio is set as zero), still up to roughly 36% of the total variability is explained (as can be seen from the red curve with corresponding $\alpha$ value in Fig. 11). Greenacre (2019) identifies the three PLRs, namely $\textrm{ln}\frac{\textrm{Si}}{\textrm{Ca}}$, $\textrm{ln}\frac{\textrm{Si}}{\textrm{Sb}}$, and $\textrm{ln}\frac{\textrm{Na}}{\textrm{Sb}}$, as being most important using a stepwise analysis. The latter two also appear to be very stable in our analysis, as they appear as nonzero in more than 30 models.

**Fig. 13:**
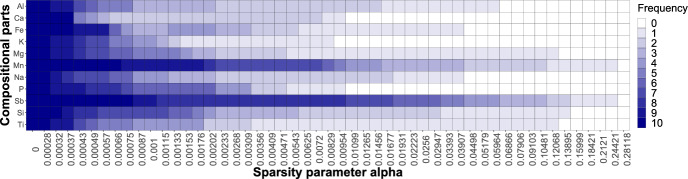
Archaeometric data: heatmap of compositional parts (in rows) for the sparse PCA solution with particular $\alpha$ (in columns). The darker the shading, the higher the occurrence of the part across nonzero PLRs

**Fig. 14:**
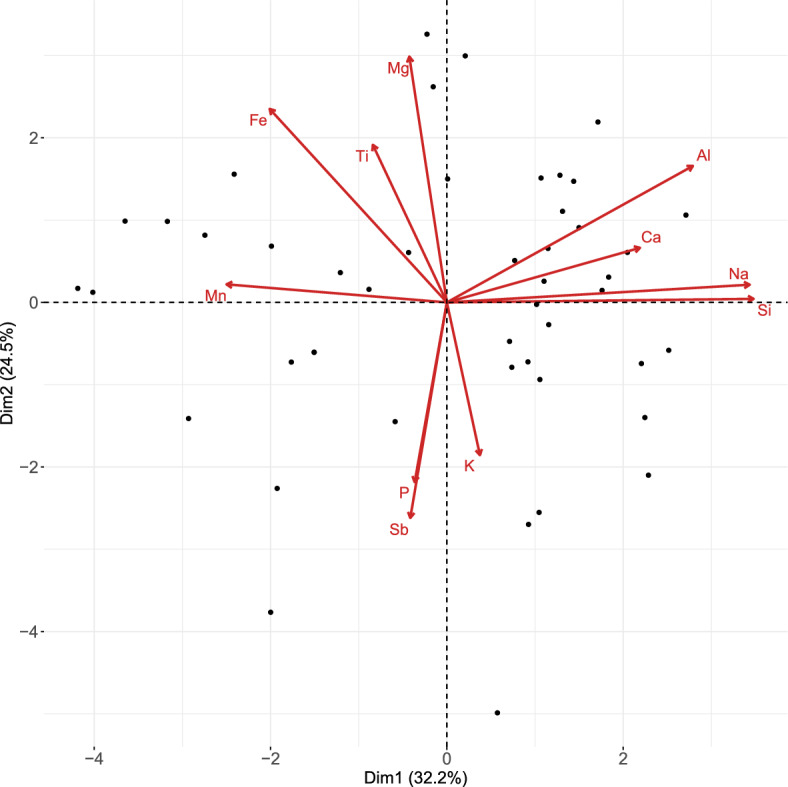
Archaeometric data: PCA biplot of the clr representation of the data

Finally, we consider the heatmap for compositional parts in Fig. 13. In particular, the elements Mn and Sb most frequently occur across selected logratios. Moreover, these parts also survive the longest, as their coloring stretches farthest to the right of the plot. These findings differ from those presented in Greenacre (2019), where the part occurring in the majority of logratios was Si. This might, however, be due rather to the non-collinearity constraint imposed on pairwise logratios, because the specific role of Si cannot be clearly observed from the corresponding compositional biplot; see Fig. 14. It can be seen that Mg, Si, and Ti also appear in nonzero logratios even in highly sparse models, albeit not as frequently as Mn and Sb. In addition, all parts occur in at least one selected PLR even in sparse PCA solutions.

## Summary and Conclusions

Compositional data analysis is based on logratio information regarding the variables. The simplest form of a logratio is in terms of the logarithm of a ratio of two variables, and this is also simple for the interpretation. The goal of this paper was thus to identify pairwise logratios (PLRs) which together explained the most relevant information contained in a compositional data set. In order to identify those PLRs, we used a sparse PCA method applied on the matrix of all possible (relevant) PLRs. Due to the sparsity of the method, some or many of the loading entries are zero, and only the nonzero entries point to important PLRs. The number of nonzero entries is determined by a sparsity parameter, and we presented a visualization where the trade-off between sparsity and explained variance can be balanced, showing the value of the corresponding sparsity parameter $\alpha$ as well. In our examples, only the first two principal components were used; in general, for more complex data, one could select the number of components in order to explain around 80% of the variance. The same plot can then still be used to find the appropriate compromise between reducing explained variance and increasing sparsity.

We presented a diverse set of simulation studies where the true important logratios were known, thereby varying the composition of relevant and noisy balances as well as the dimension *D* of the composition to gain a good idea of the sensitivity of the proposed sPCA method across various scenarios. The proposed methodology turned out to be successful in identifying those PLRs. We compared our method with the STEP algorithm (Greenacre 2019), which is designed to represent the data set in a new coordinate system (without dimension reduction) which is built upon PLRs. When comparing our proposed method with STEP, we focused on (i) the methods’ ability to identify important PLRs, (ii) their computing time, and (iii) the amount of data they could handle. Regarding (i), sPCA was, overall across the considered simulation scenarios, more successful in identifying the true important PLRs than STEP. The discrepancy between the two was greatest in the scenarios with the fewest relevant balances and larger *D*. The graphical tools introduced, which are based on explained variability and sparsity, can further assist practitioners in effectively analyzing CoDa to this end. STEP, unlike sPCA, may be used to construct a logratio coordinate system, but in doing so, the choice of PLRs is limited, whereas sPCA considers all pairwise logratios. Regarding (ii), STEP is faster than sPCA in lower dimensions (small *D*) but is outperformed by sPCA in higher dimensions, as the computation time of the former increases more steeply with the dimension *D* of the composition. Regarding (iii), sPCA proved capable of handling compositions with a greater number of parts, even as many as 100. Investigating the performance of sPCA with hundreds or more parts would be an interesting task for future research.

In our empirical applications, we present several graphical tools to aid practitioners in their multivariate analysis with CoDa. Next to the already mentioned plot to select the sparsity parameter, we provide a visualization showing stability paths for the PLRs. They reveal which PLRs remain in the PCA model if the sparsity of the PCA solution gradually increases. The important, stable logratios highlighted by our analysis also turned out to be important in previous CoDa analysis on the considered geochemical data sets. Finally, we provide heatmaps for the original compositional parts, thereby permitting one to evaluate their importance for the multivariate analysis.

In this paper, we focus on PCA, which remains a workhorse method for multivariate data analysis with CoDA. The paper thereby offers a clear way to become oriented in the use of many PLRs in PCA, even for compositions with a moderate number of components. As the recent trend in CoDa analysis is a preference for simple PLRs over more sophisticated logratio coordinate representations, the use of sparse models with few PLRs promises to be of increasing importance beyond the scope of PCA as well, for instance, to three-way compositional data analysis (Di Palma et al. 2018) or classification tasks. Finally, a limitation of PCA is, however, that it is confined to linear relationships among the variables. If the relationships are nonlinear, then PCA may not be able to capture them effectively. It would be interesting to further investigate the potential for using machine learning methods instead for exploratory data analysis, although care is needed to ensure the appropriate treatment of the compositional nature of the data.

## Figures and Tables

**Fig. 15:**
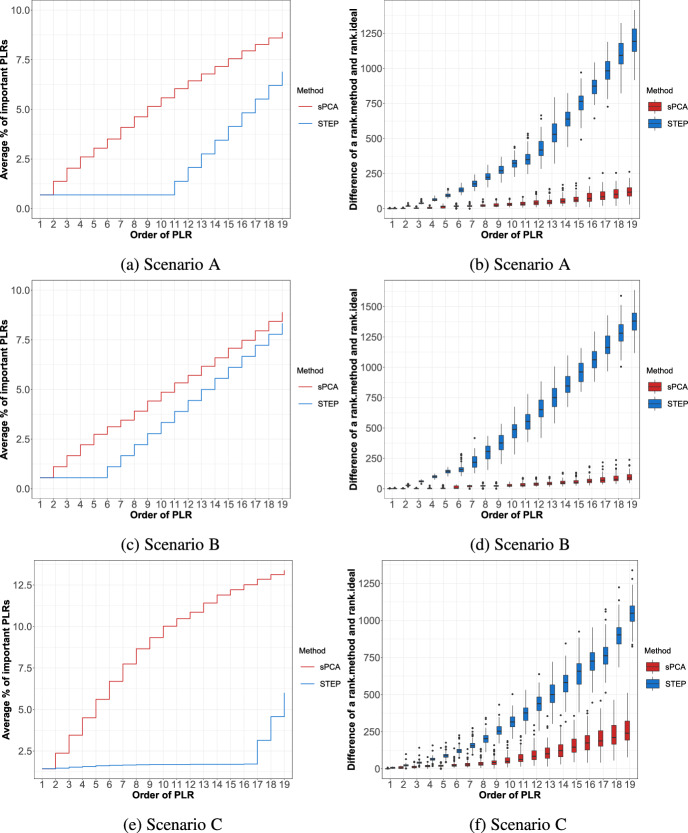
Comparison of sparse PCA with STEP on simulation scenarios A, B, and C for $D=20$. Left: average percentage of correctly identified PLRs for sPCA (red) and STEP (blue) for the first $D-1$ ordered PLRs (PLR) on the horizontal axis. Right: boxplots showing the differences between the cumulative ranks of the PLR obtained from sPCA (red) or STEP (blue) and the ideal ranks

**Fig. 16:**
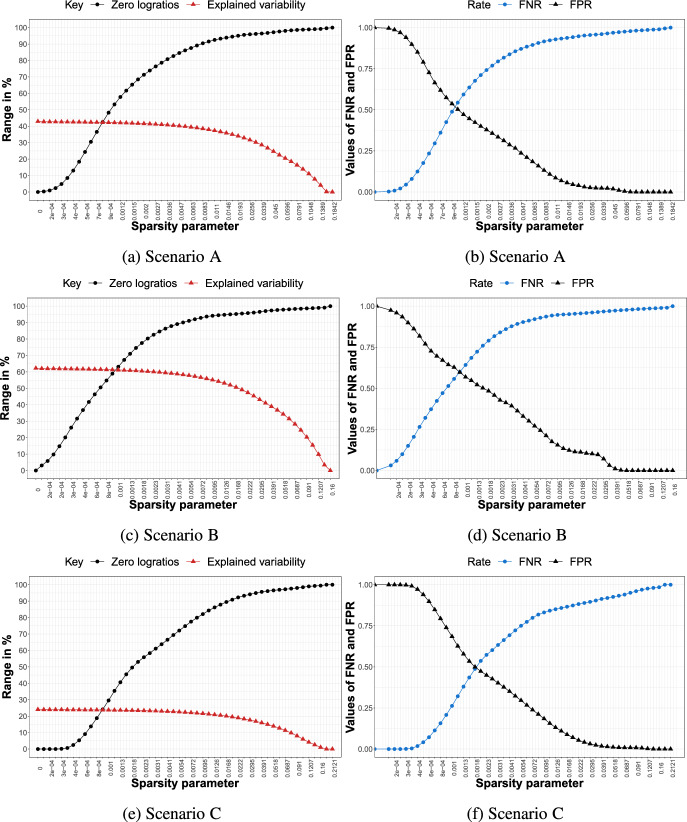
Simulation scenarios **A**, **B**, and **C** for $D=20$. Left: percentage of zero logratios (black) and explained variability (red) for different values of the sparsity parameter $\alpha$. Right: FPR (black) and FNR (blue) for different values of $\alpha$

## Appendix A: Results for the Different Simulation Scenarios with $D = 20$

The following section shows simulation results for all three simulation scenarios A, B, and C with $D = 20$ parts. The data are simulated using the procedure described in Sect. 3, considering 10 (scenario A), 15 (scenario B), and four (scenario C) relevant balances.

When comparing sparse PCA and STEP via (i) the percentage of important PLRs and (ii) the rank of the chosen logratios, it can be seen in Fig. 15 that sparse PCA outperforms STEP on both criteria. Unlike the results for $D=10$, the advantage of sparse PCA over STEP is now also more apparent in simulation scenario C.

Next, we zoom in to the results of sparse PCA in Fig. 16. In scenario A, a lower percentage of explained variability is obtained for the dense model (compared to the $D=10$-part case), but it again decreases slightly with increased sparsity. The sparsity level rises even more rapidly than in the $D=10$-part case, achieving around 53% of zero logratios ($\alpha = 0.001$) with roughly 42% of explained variability. Similarly in scenario B, the sparsity level shoots up quickly when the sparsity parameter increases. Finally, for scenario C, we observe a similar trend (as for the $D=10$-case) in the decrease in explained variability with increasing sparsity.
